# A bimolecular fluorescence complementation flow cytometry screen for membrane protein interactions

**DOI:** 10.1038/s41598-021-98810-2

**Published:** 2021-09-28

**Authors:** Florian Schmitz, Jessica Glas, Richard Neutze, Kristina Hedfalk

**Affiliations:** Department of Chemistry and Molecular Biology, Gothenburg University, Box 462, 405 30 Göteborg, Sweden

**Keywords:** Biochemistry, Membrane proteins, High-throughput screening, Protein design

## Abstract

Interactions between membrane proteins within a cellular environment are crucial for all living cells. Robust methods to screen and analyse membrane protein complexes are essential to shed light on the molecular mechanism of membrane protein interactions. Most methods for detecting protein:protein interactions (PPIs) have been developed to target the interactions of soluble proteins. Bimolecular fluorescence complementation (BiFC) assays allow the formation of complexes involving PPI partners to be visualized in vivo, irrespective of whether or not these interactions are between soluble or membrane proteins. In this study, we report the development of a screening approach which utilizes BiFC and applies flow cytometry to characterize membrane protein interaction partners in the host *Saccharomyces cerevisiae*. These data allow constructive complexes to be discriminated with statistical confidence from random interactions and potentially allows an efficient screen for PPIs in vivo within a high-throughput setup.

## Introduction

To understand cellular networks that underpin normal cell function requires the robust identification of protein–protein interactions (PPI). PPIs participate in critical functions such as signalling, posttranslational modification, trafficking and environmental communication. When functional interactions are disrupted, this can lead to autoimmune diseases and cancer^1^. Membrane proteins and their PPI networks are common drug targets^2^, which adds emphasis to the need for better characterization of their PPIs. Aquaporins are a sub-family of integral membrane proteins that facilitate the passive transport of water across a biological membrane while preserving a transmembrane proton gradient. Aquaporin malfunction can lead to the development of a wide range of medical conditions including neurological disorders^3–5^, auto-immune diseases^6^ and inflammatory related responses^7,8^. Importantly, to explore the involvement of aquaporins in diseases, the understanding their regulatory processes by PPIs is crucial. A recent example is the interaction between aquaporin 4 (Aqp4), a human aquaporin mainly expressed in astrocytes, and calmodulin (CaM) which has been shown to mediate re-localization of Aqp4 to the cell surface leading to cytotoxic edema, a condition of uncontrolled water influx that commonly occurs after injury^9,10^.

Several methods have been developed to detect PPIs, all of which are based upon different consequences of these interactions^11^. These techniques include Surface Plasmon Resonance^12^, Two-Hybrid methods^13^, and biochemical approaches such as Co-Immunoprecipitation^14^ and Pull-down assays^15^. Irrespective of whether or not the detection method is based upon biophysical, genetic or biochemical foundations, it is always necessary to assess whether the potential benefits of a given approach outweigh the method’s inherent disadvantages. More recently, a variety of methods for surveying the interactome have emerged^11,16^, of which fluorescence-based techniques such as fluorescence resonance energy transfer (FRET) and Bimolecular fluorescence complementation (BiFC) for screening of PPIs in living cells^17,18^ have attracted attention. BiFC can be readily performed in different host systems ranging from plant cells^19^ to more complex mammalian cells like HEK293^20^. Complementation fluorescence was initially quantified using fluorescence microscopy^21^. However, the benefits with flow cytometry for the analysis of fluorescence intensity became apparent enabling high throughput screening while avoiding artefacts arising from manual readout^22^. The detection of PPIs using BiFC pairing has intrinsic challenges such as unspecific fluorescence background signal due to self-assembly of the fluorophore partner, and possible tethering of BiFC partners during translation and translocation^23^. Thus, while BiFC is a highly attractive option, when using BiFC it is essential to keep awareness of these limitations^24^.

In a previous study we established the BiFC method to validate the direct PPIs between the tetrameric complex formed by human aquaporin 0 (hAQP0) alone, and with the regulatory protein Calmodulin (CaM). Each protein target was attached to a complementary YFP molecule and both yielded BiFC complexes when expressed within the yeast host *Saccharomyces cerevisiae*^25^. This process was driven by the specific interaction between aquaporin molecules, or between CaM and the C-terminus of hAQP0^26^ which brought the YFP fragments into close proximity. The maturation of YFP was followed by the fluorescence read-out, providing a method for membrane protein complex screening whereby the in vivo interaction can be analysed directly within the expression host membrane. The formation of PPI complexes was independently confirmed by Native Page^25^. Here we further develop this screening procedure by applying population screening and quantification of the BiFC complex using flow cytometry, thereby establishing a complete procedure from the transformation and growth of cells to the analysis of the fluorescent signal. This protocol facilitates a higher throughput relative to traditional, labour intensive approaches and could be utilized to screen for novel PPIs.

## Results

### Expression of aquaporin BiFC constructs in *S. cerevisiae*

**Figure 1:**
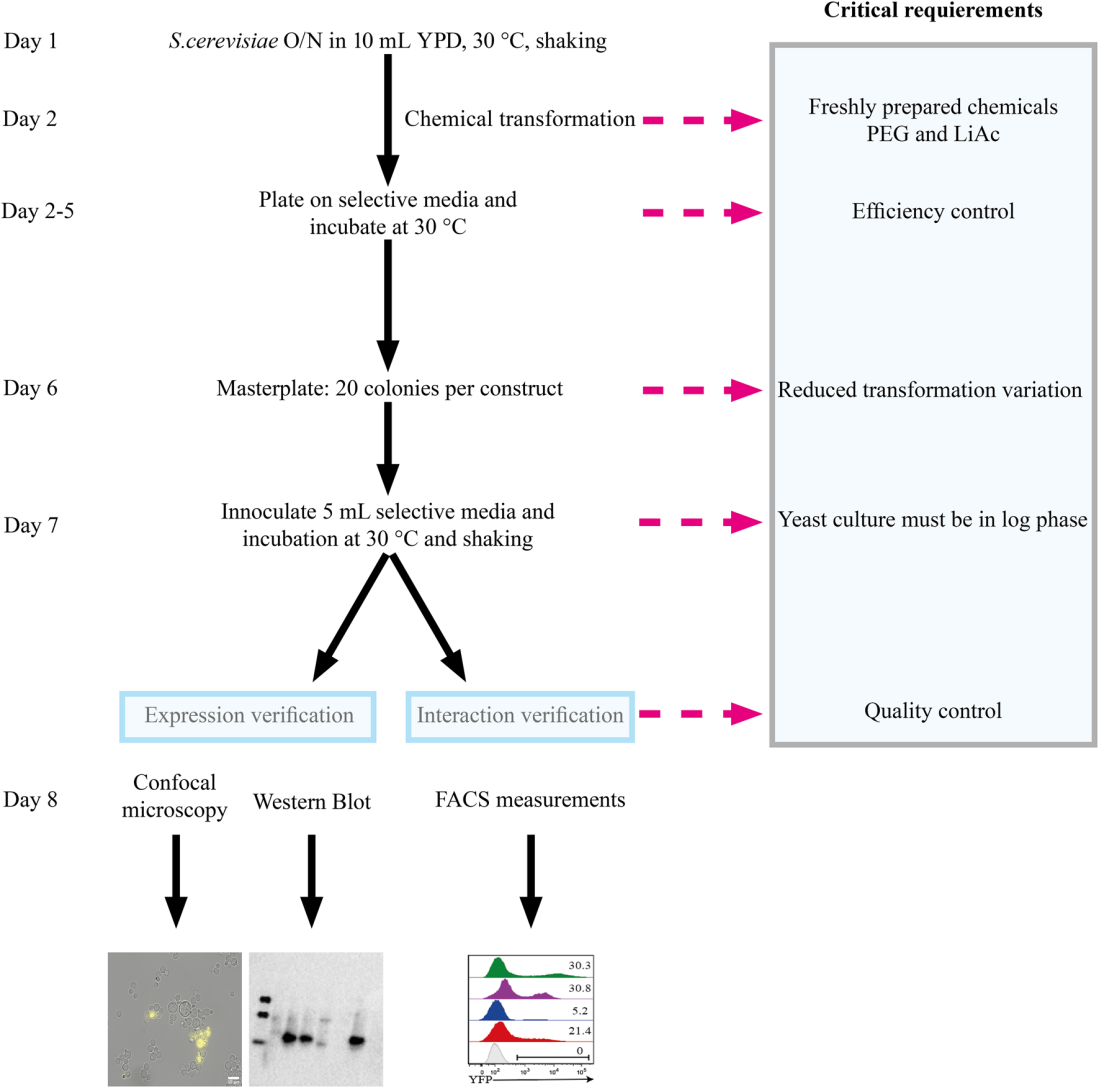
Experimental design of a standardized cell preparation for expression and interaction analysis of BiFC complexes produced in *Saccharomyces cerevisiae* cells. The schedule shows that the whole screening process is completed in less than 14 days, emphasizing critical steps.

We analysed the expression and interactions of Aqp0 using the experimental design summarized in Fig. 1. All samples show an intrinsic variation in the fluorescence of the transformants when measured using flow cytometry. To minimize this effect, fluorescence cytometry was used as a pre-check on ten samples taken directly from the growth plate to evaluate the transformation efficiency. If fluorescence was detected from these colonies, the transformation was considered successful and twenty colonies were then picked and transferred to a fresh synthetic complete (SC) media agar plate. This procedure was performed for a minimum of three biological replicates. Ten colonies from each transformation were then grown in liquid media to their log phase and analysed using flow cytometry. As negative controls, transformants containing only one plasmid of the BiFC pair were treated the same way.

**Figure 2:**
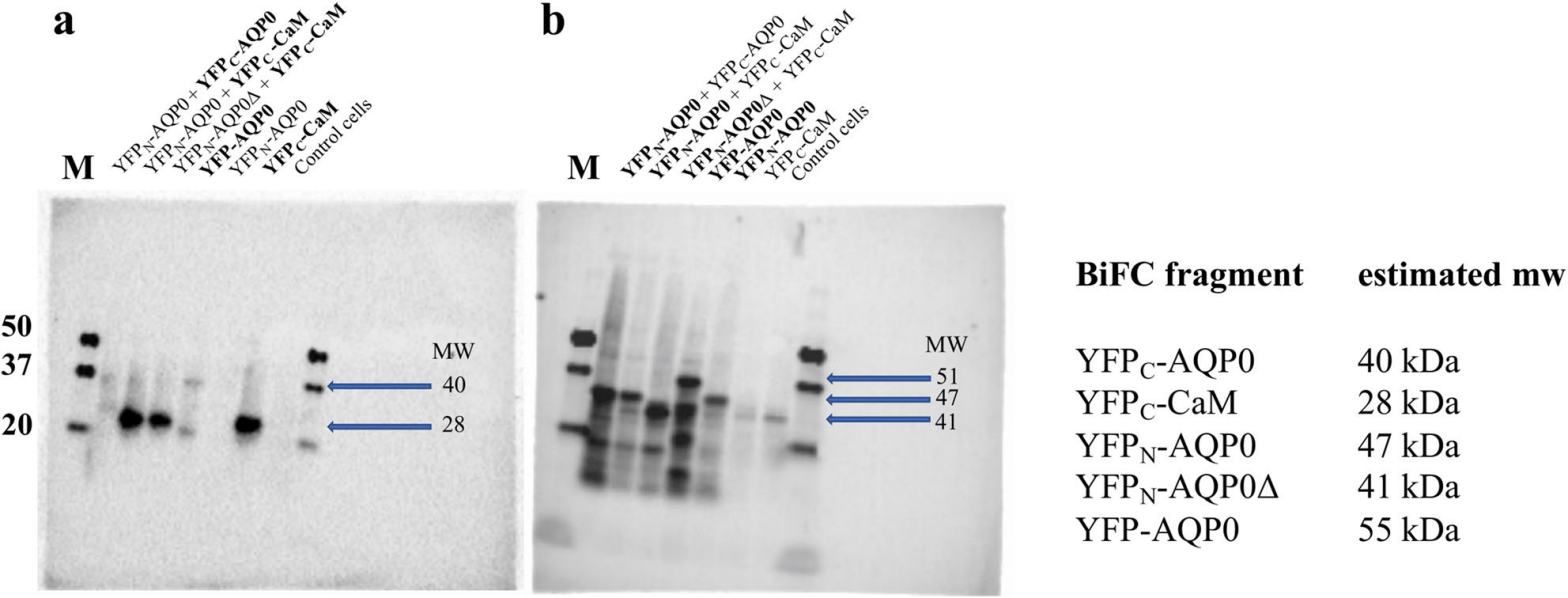
Immunoblot analysis of the various BiFC complexes obtained from cell lysates. Samples were applied in the following order on the blots (a and b): (M) Marker, molecular weight in kDa, (1) YFP_N_ − AQP0 + YFP_C_ − AQP0, (2) YFP_N_ − AQP0 + YFP_C_ − CaM, (3) YFP_N_ − AQP0∆ + YFP_C_ − CaM, (4) YFP − AQP0, (5) YFP_N_ − AQP0, (6) YFP_C_ − CaM, (7) non-transformed *S. cerevisiae* cells. Monoclonal antibodies against YFP_C_ (a) or YFP_N_ (b) were used. The immunoblot confirms similar expression levels of the BiFC components. The bands corresponding to the BiFC fragments are indicated by arrows with the expected molecular weight and the part of the BiFC complex detected by the antibody is highlighted in bold.

To confirm proper production of individual entities of the BiFC-pair, standardized high density cell lysates of overnight cultures were separated by SDS-PAGE and analysed by immunoblot using anti-YFP split variant (YFP_C_, YFP_N_) antibodies (Fig. 2). Although evidence for BiFC complexes could also be detected using this analysis, the major immunoblot signals correspond to individual parts and show an excess of expressed fragments relative to the BiFC complexes. Thus, immunoblot analysis verifies the production of all of the BiFC components when expressed within the host, and provides evidence that a small fraction of the expressed protein successfully forms mature BiFC complexes.

### Mature BiFC complexes visualized by fluorescence microscopy

**Figure 3:**
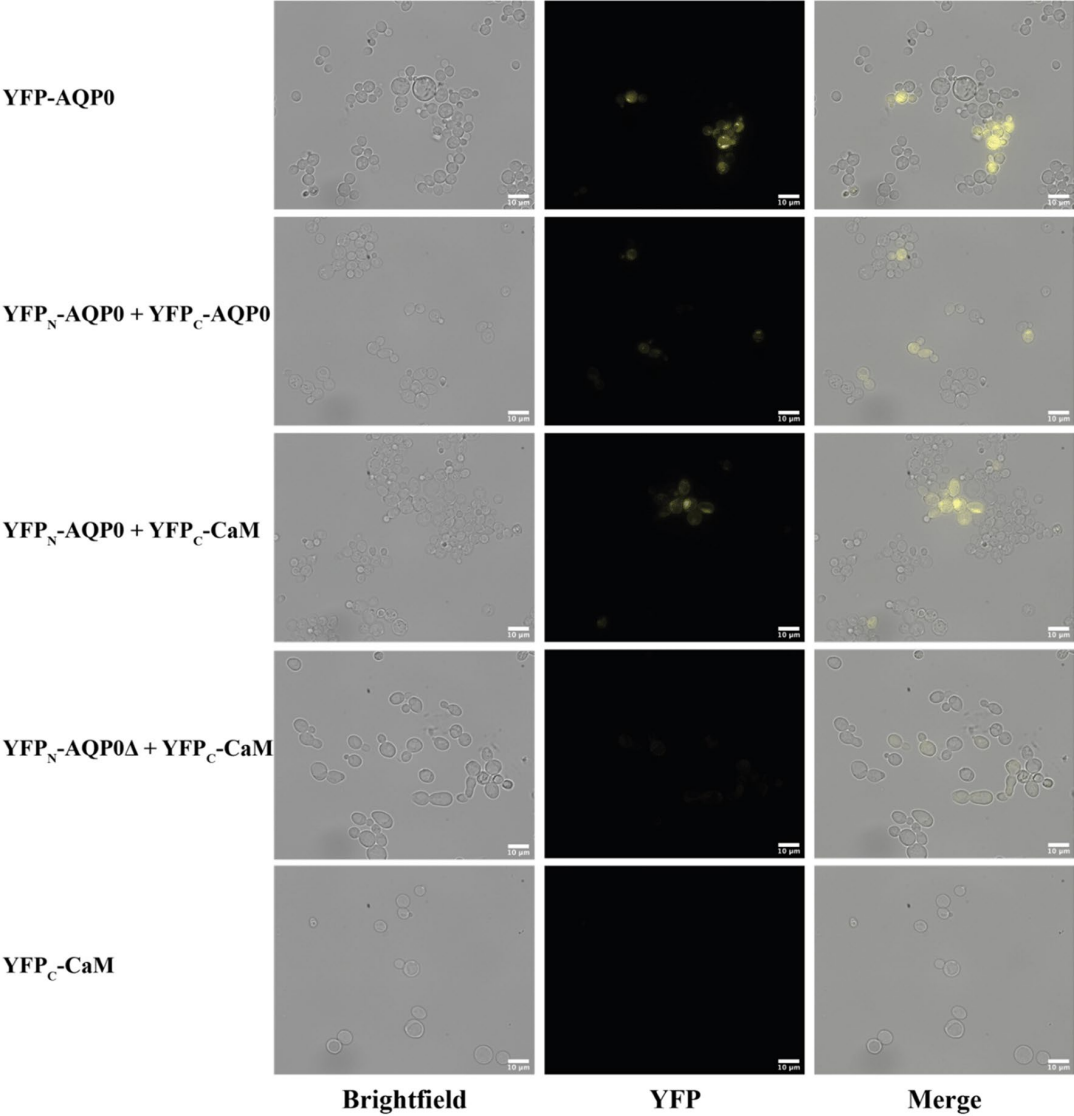
Bright field and fluorescence images of subcellular localisation of BiFC complexes produced in *S. cerevisiae* cells. Highest fluorescence signal was obtained from the positive control, cells producing the full length chromophore fused to human aquaporin 0, YFP − AQP0. Cells transformed with interaction complexes YFP_N_ − AQP0 + YFP_C_ − AQP0 and YFP_N_ − AQP0 + YFP_C_ − CaM, respectively, showed an intermediate signal, while very faint fluorescence was observed for the C-terminus deficient BiFC formation control, YFP_N_ − AQP0∆ + YFP_C_ − CaM. Cells expressing single plasmid were used as non-fluorescent controls.

Intracellular production of mature BiFC complexes and the absence of fluorescent aggregates was confirmed using fluorescence microscopy. As was previously observed^25^ the most intense fluorescence signal was detected from the single plasmid transformant YFP − AQP0 (Fig. 3). By comparison, BiFC complexes requiring co-transformation (YFP_N_ − AQP0 + YFP_C_ − AQP0 and YFP_N_ − AQP0 + YFP_C_ − CaM) yielded lower fluorescence intensities, which is highly intuitive since those constructs required that a complex formed between the co-expressed proteins and physical contact between both YFP fragments. Both the single plasmid control (YFP_N_ − AQP0) and the C-terminus deficient BiFC complex formation control (YFP_N_ − AQP0∆ + YFP_C_ − CaM) showed extremely low fluorescence intensities, supporting the hypothesis that fluorescent YFP will not form if the CaM binding site of AQP0 is deleted. In addition to qualitative confirmation of fluorescence yields, brightfield fluorescence microscopy also provides information concerning the overall fitness of yeast cells since abnormal cell shapes may indicate stress.

### Harvesting cells during log phase growth aids cytometry analysis

**Figure 4:**
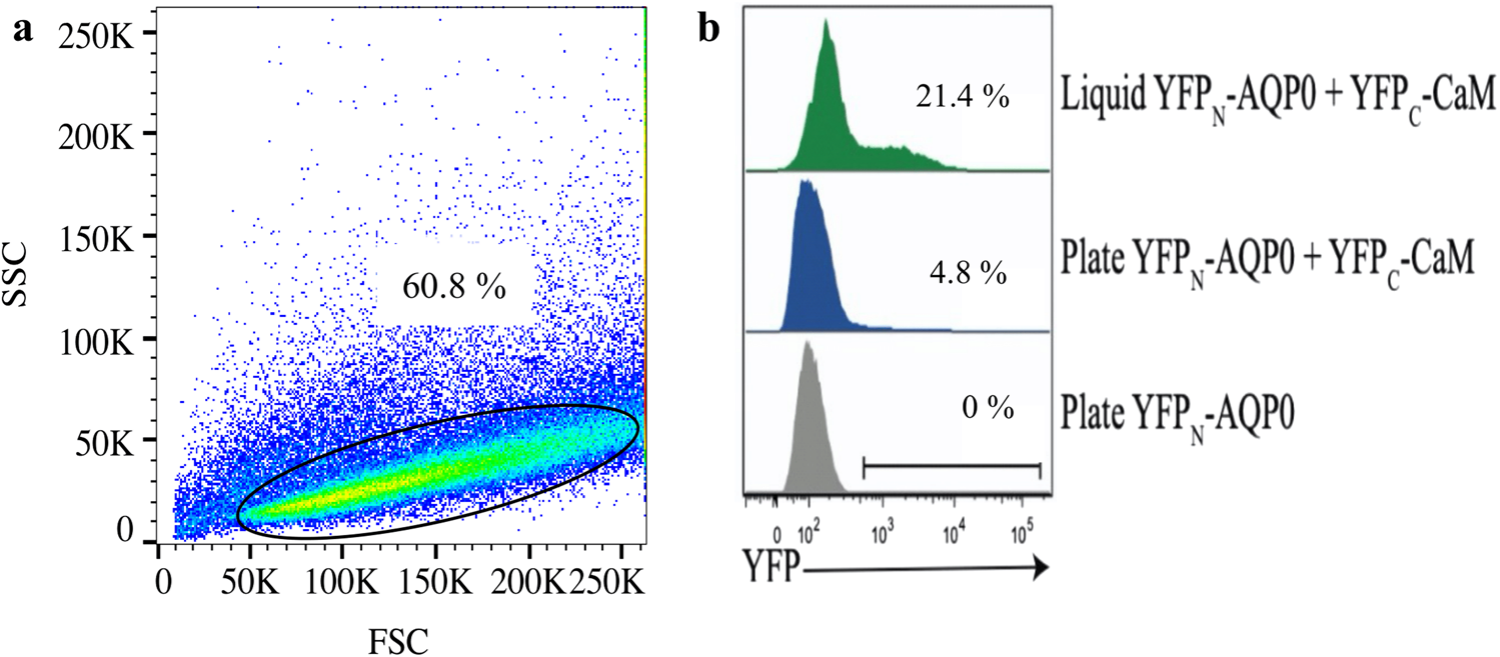
Flow cytometry quantification of BiFC yields. (**a**) An exemplary pseudo-color plot of Aqp0 − CaM complex illustrating 100.000 events. A standardized gate, visualized as a sloping ellipse, selects individual yeast cells for analysis. 60.8% of all events were recorded within the selected domain. Side scatter (SSC on the y-axes is an indicator of complexity and forward scatter (FSC) on the x-axes is an indicator of size. (**b**) Comparison of the Aqp0 − CaM complex grown in SC liquid culture (OD_600_ = 0.5) with the one grown on SC media plates. The fluorescence signal was evaluated from a threshold value of 500 intensity units.

When analysing the formation of BiFC complexes by flow cytometry, a gate is required that selects individual intact yeast cells. Figure 4a provides an example of this procedure using the Aqp0-CaM BiFC complex formed when YFP_N_-AQP0 and YFP_C_-CAM come together in the cell. The selected domain is indicated within a sloping ellipse and 60.8% of all events were recorded within this gate. This analysis also revealed that a higher fraction of cells containing a mature BiFC complex formed when pre-selected fluorescent cultures are grown to the log-phase (OD_600_ = 0.5) rather than when picking yeast cells directly from the SC agar plate. Specifically, the fraction of recorded events achieving a fluorescence yield above 500 fluorescence intensity units, which is the threshold to distinguish auto fluorescence from the YFP signal, was approximately four times higher for the Aqp0 − CaM complex grown in liquid cultures rather than on plates (21.4% versus 4.8%, Fig. 4b). We therefore conclude that cultivating transformed cells in liquid cultures and harvesting these during their log phase aids the quantification of the cytometry fluorescent cells lines. This procedure was used throughout the remaining analysis.

### Quantification of BiFC yields using flow cytometry

**Figure 5:**
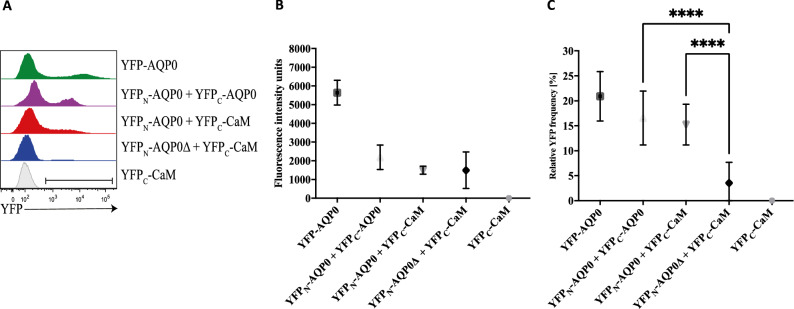
Quantification of BiFC yields using flow cytometry. (**A**) Analysis of constructs compared in this study together with the single plasmid negative control (YFP_C_ − CaM). The histograms show the distribution of fluorescence intensities in the log phase of growth. The fluorescence signal was evaluated from a threshold value of 500 intensity units, discriminating the YFP-signal from auto fluorescence. (**B**) Average fluorescence intensity is shown for each complex together with the standard error of the mean (n ≥ 10 technical repeats based on at least three biological repeats, i.e. independent transformation events, see Table 1). (**C**) The average of the fraction of fluorescent cells in percentage is shown for each complex together with standard error of the mean (n ≥ 10 technical repeats based on at least three biological repeats, i.e. independent transformation events). An unpaired two component t-test with Welch’s correction was used to determine statistical difference of fluorescence frequency between YFP_N_ − AQP0 + YFP_C_ − CaM and YFP_N_ − AQP0∆ + YFP_C_ − CaM as well as YFP_N_ − AQP0 + YFP_C_ − AQP0 and YFP_N_ − AQP0∆ + YFP_C_ − CaM with *p* values < 0.0001 demonstrating a statistically robust discrimination above a certain yield, as indicated ****.

With conditions established for robust flow cytometry studies, we analysed the intensity and frequency at which a specific number of fluorescence counts occurred within a certain range of events for each of four constructs: YFP − AQP0; YFP_N_ − AQP0 + YFP_C_ − AQP0; YFP_N_ − AQP0∆ + YFP_C_ − CaM; and YFP_C_ − CaM, where AQP0∆ has the C-terminus removed (Table 1). The distribution of fluorescence intensity from the different constructs is illustrated by a histogram example for each construct, where the first peak is showing the auto fluorescence of all cells below the threshold value (500 fluorescence intensity units) and the second peak shows the distribution of all cells above the threshold value (Fig. 5a). Of those events which showed fluorescence, the intensity of this fluorescence signal was strongest for the positive control YFP − AQP0 at 5600 ± 700 fluorescence units (Fig. 5b). This is indicated by the peak of the distribution being shifted to higher count-rates relative to those recorded for other samples (Fig. 5a) which is highly intuitive since this was the only construct to contain the full-length YFP, whereas all other constructs required two YFP fragments to be brought into close proximity prior to YFP maturation. In comparison, the second peak of YFP_N_ − AQP0 + YFP_C_ − AQP0 and YFP_N_ − AQP0 + YFP_C_ − CaM is located further to the left where the peak of YFP_N_ − AQP0 + YFP_C_ − AQP0 is showing a clearer separation from the auto fluorescence peak than YFP_N_ − AQP0 + YFP_C_ − CaM visualizing as stronger distribution of cells with a higher average fluorescence intensity signal. Very few cells of the YFP_N_ − AQP0∆ + YFP_C_ − CaM complex display a fluorescence signal above auto fluorescence, resulting in a flat peak in comparison to the constructive BiFC complexes. However, the width of the peak indicates that the rare YFP maturation event leads to a similar fluorescence signal, which can be confirmed by Fig. 5b.

**Table 1:** Experimental setup for the evaluation of fluorescence intensity and relative YFP frequency of BiFC complexes.

Constructs	Biological repeats	Technical repeats	Range of analysed events
YFP − AQP0	3	31 (10 + 11 + 10)	29,000–68,000 (average 53,000)
YFP_N_ − AQP0 + YFP_C_ − AQP0	3	32 (10 + 11 + 11)	17,500–67,000 (average 36,500)
YFP_N_ − AQP0 + YFP_C_ − CaM	3	33 (11 + 12 + 10)	16,500–82,000 (average 44,500)
YFP_N_ − AQP0Δ + YFP_C_ − CaM	3	44 (13 + 19 + 12)	15,500–82,000 (average 40,000)
YFP_C_ − CaM	3	30 (10 + 10 + 10)	71,000–78,000 (average 75,000)

Number of biological repeats (independent transformation event) and technical repeats are shown for each construct. The range and average of events analyzed in each measurement are displayed.

The fraction of events yielding more than 500 fluorescence counts per event were then calculated from these data. Both positive controls, YFP − AQP0 and YFP_N_ − AQP0 + YFP_C_ − AQP0, gave sizeable populations of 21% ± 5% and 17% ± 5% respectively of cells displaying a fluorescence above this threshold. *S. cerevisiae* cells expressing both YFP_N_-AQP0 + YFP_C_-CaM constructs also showed fluorescence above 500 counts per event in 15% ± 4% of the selected cells, slightly lower than that for the YFP_N_ − AQP0 + YFP_C_ − AQP0 control. By contrast, the YFP_N_ − AQP0∆ + YFP_C_ − CaM constructs gave a much lower yield of 4% ± 4% for the fraction of events to show fluorescence above this threshold (Fig. 5c). The remaining single plasmid transformants (YFP_C_ − CaM and YFP_N_ − AQP0) which were included in the flow cytometry evaluation as negative controls, did not yield a significant fraction of events (< 0.1%) with fluorescence above threshold.

In summary, both constructive BiFC pairs show similar average fluorescence intensities, with the YFP_N_ − AQP0 + YFP_C_ − AQP0 recording an average fluorescence intensities per event above threshold of 2200 ± 700 fluorescence units and the corresponding value for YFP_N_ − AQP0 + YFP_C_-CaM being 1500 ± 200 fluorescence units (Fig. 5b). Likewise, the YFP_N_ − AQP0∆ + YFP_C_ − CaM construct yields a similar value of 1500 ± 100 fluorescence units suggesting that these complexes, while relatively rare, fluoresce as much as a constructive BiFC complex but with a substantially higher variation. The control single plasmid transformants YFP_C_ − CaM, on the other hand, did not show any analysable fluorescence fraction of events. Thus, similar fluorescence intensity of the constructive BiFC complexes YFP_N_ − AQP0 + YFP_C_ − AQP0, YFP_N_ − AQP0 + YFP_C_ − CaM and the construct YFP_N_ − AQP0∆ + YFP_C_ − CaM suggests that any complementation of the YFP molecule event results in a similar strong fluorescence signal. However, these events occur much less frequently in proteins that do not interact, and as a consequence the fluorescence frequency value has been shown to be the more accurate evaluation for detecting true interactions.

## Discussion

Diverse host systems have been developed to support BiFC studies in prokaryotic cells^27^ eukaryotic yeast systems^28–30^ and human cell lines^31^. BiFC has previously been used to detect human aquaporin interaction partner in vivo^25,27^ and to confirm aquaporin PPIs in living plant cells^32,33^. We previously developed a method for identifying PPIs by laboriously examining hundreds of fluorescence microscopy images^25^. The integration of flow cytometry readout for BiFC studies provides a step towards higher throughput screening of molecular PPI processes, both in human cells^34^ as well as in yeast cells^29,30^.

In this work we show how flow cytometry can reliably identify a validated PPI of an integral membrane protein when both proteins are expressed in *S. cerevisiae*. This development removes a subjective and time-consuming step of a skilled researcher examining hundreds of images by eye and therefore has the potential to both achieve considerably higher throughput and to provide more quantitative foundations for the method. While BiFC complementation is an established method for the detection and validation of PPIs, it has intrinsic disadvantages of it being challenging to distinguish between background signal and valid molecular interactions leading to potential false-positives. The development of split fluorophores has improved signal-to-noise ratios^35^, although it is common to report only the fluorescence intensities of complementation events. In this work we rationalized growth conditions prior flow cytometry measurements and this reduced unspecific BiFC background, providing a robust protocol that may allow unknown PPI interactions to be detected in a medium throughput manner (Table 1). Moreover, flow-cytometry analysis itself leads to quantification and statistically robust criteria emerge for identifying PPIs above background, with the proportion of cells displaying fluorescence events above threshold being, in this case, a more powerful criteria discriminating PPIs from false positives. In comparison to other studies, where the interaction between aquaporin isoforms and CaM has been verified by Microscale Thermophoresis (MST)^9,26^, BiFC complementation has the advantage of enabling quantification and screening of interactions in vivo and does not require prior purification of the proteins, which can be especially challenging for membrane proteins.

Furthermore, worth considering is that the distance and mutual orientation of the complexes can have a critical impact on the constructive BiFC pair formation. Therefore, it is important to test different combinations of construct design to find the optimal pair, as the way the fluorophore fragment and the protein of interest are combined plays a role in the efficiency of complex formation^25^. In addition, the correct construct design is also influenced by the choice of the flexible linker sequence between the YFP fragment and the protein of interest, as the linker length and the specific sequence have an influence on the expression, activity and localization of expressed proteins and their complexes^36,37^. In a further study, we have compared the YFP signal using two different linkers for the constructive BiFC complex YFP_N_ − AQP0 + YFP_C_ − CaM. Interestingly, using the longer linker, we obtained an enhancement in the YFP frequency signal but not in YFP intensity from the flow cytometry analysis (Supplementary Fig. 1). The length of the linker therefore also seems to have an influence on how likely it is that the YFP fragments mature into a complex. Another critical aspect for the construct design in the BiFC-evaluation is the inclusion of proper negative controls, where the most valid control is a non-constructive complex of the specific pair based on mutated proteins hindering the interaction^38^.

While the flow cytometry analysis demonstrated here satisfactorily identified fluorescent complexes, fluorescence imaging is a valuable complement in order to assure healthy cells are included in the analysis, and we recommend that the quality of the cells is checked for each designed construct. In particular, when using BiFC to study membrane protein complexes, the membrane localization of the complex can be confirmed and constructs leading to internal protein aggregates rejected. Moreover, some mutations that may be included as control studies can be complementary at the molecular level but may impact the overall health of the cells, yet this potential problem should be easily identified using confocal fluorescence microscopy.

In conclusion, we present an approach that combines qualitative and quantitative criteria for the screening of PPIs by combining BiFC, fluorescence imaging and flow cytometry. The relative frequency of cells showing fluorescence above a specified threshold is able to identify constructive complex formation with high statistical confidence (Fig. 5) while reducing the risk of false positives during screening for novel PPIs. We suggest that this approach could be further developed to allow high-throughput cDNA library screening of novel interaction PPIs^39^ by the use of fluorescence activated cell sorting (FACs) to sort out individual, promising candidates on the basis of their fluorescence and thus identify possible interaction partners. Here, the choice of yeast replication plasmids, as used in this particular study, is crucial as it ensures the possibility of losing the unwanted plasmid from the cell through selection pressure. This is especially important in the development of the method using fluorescence activated cell sorting (FACS) to identify individual promising interaction partners based on fluorescence.

Should this vision be realizable, this approach may provide a valuable tool for gleaning new information on how membrane proteins interact with other proteins in healthy cells and could potentially assist in understanding when cellular interaction pathways dysfunction and lead to diseases.

## Methods

### Genes, vectors, and strains

There is no direct involvement of human participants in the study. The CaM gene was kindly provided by Rachel Klevit (University of Washington) and the human AQP0 gene, codon optimized for production in yeast, was purchased from Genscript (Piscataway, NJ). The BiFC pairs, including human AQP0 and CaM, were cloned in the p423GPD and p426GPD vectors and transformed to *S. cerevisiae* (MYA-1662™ his3, ura3-52), as previously described^25^. For this specific study the following constructive constructs were selected for evaluation by flow cytometry screening; YFP − AQP0, YFP_N_ − AQP0 + YFP_C_ − AQP0, YFP_N_ − AQP0 + YFP_C_ − CaM, YFP_N_ − AQP0∆ + YFP_C_ − CaM, YFP_N_ − AQP0, YFP_N_ − AQP0 and YFP_C_ -CaM.

### Chemical transformation of *S. cerevisiae*

For transformation of plasmids, *S. cerevisiae* cells with HIS or HIS/URA deficiency selection marker (MYA-1662™ strain) were inoculated in 5 ml YPD medium and grown overnight at 30° C while shaking. The next day, the cells were inoculated in 50 ml YPD medium to a starting OD_600_ of 0.25 and grown at 30° C to reach an OD_600_ between 0.7 and 1.0. For the chemical transformation, cells were fractionated into 50 µl batches and for a single plasmid transformation, each batch was mixed with 240 µl freshly prepared PEG4000 (50%), 36 µl 1 M freshly prepared LiAc, 50 µg freshly denatured salmon sperm, 1–2 µg (maximum 10 µg) of plasmid DNA and mQ water was added to a final volume of 360 µl. In case of double plasmid transformation, a minimum of 2 µg (maximum 10 µg) of each plasmid DNA must be used. The mixture was incubated at 30° C for 30 min with shaking and a subsequent heat shock followed for 25 min at 42° C. The cells were spun down for 15 s at 5.500 g. The pellet was resuspended in 200 µl mQ water, transformed cells were plated out on selective SC-agar plates and incubated at 30° C. The single colonies were grown for at least three days, to become fully visible on the SC-agar plates.

### Growth of transformants producing the BiFC complexes

Twenty colonies from each transformation were picked and transferred to a fresh SC media agar plate (Masterplate) and grew overnight at 30 °C. For each BiFC construct and control, twelve colonies were regrown overnight in 5 ml SC medium at 30° C with shaking. The criterion for the selection of the twelve colonies chosen was the amount of material provided by each colony to successfully start a preculture. The following day, the cells from the overnight culture were diluted to OD_600_ = 0.2 into a 12-well tissue culture plate and incubated at 30° C while shaking to reach an OD_600_ of 0.5, assuring a generation time of two hours being a quality marker for a culture of healthy cells. Cultures having a prolonged generation time were not included in the fluorescence analysis.

### Fluorescence readout using flow cytometry

Fluorescence intensities of the cell samples were evaluated using a FACSMelody (BD) flow cytometer (100 µm nozzle size, blue excitation laser at 488 nm). For the fluorescence measurements, 500 µl sample, cell culture of OD_600_ = 0.5, was loaded into the FACS machine assuring a good resuspension of the cells. Debris and non-uniform cells were excluded and the fluorescence intensity of 100.000 cells of the live population was excited at 488 nm and evaluated on the FITC channel (527/32 nm bandpass filter). A threshold was set at 500 fluorescent intensity units to distinguish between autofluorescence and YFP signal.

### Immunoblot analysis

To confirm equal expression levels of the BiFC constructs, cell lysates were evaluated using Immunoblot analysis. In brief, the cells of an overnight culture of the different constructs were spun down (5.500 g, 5 min.) and diluted in PBS to an OD_600_ of 5. Each sample was resuspended in 200 µl 1 × SDS resuspension buffer (46 μl 4 × Laemmli sample buffer (BioRad), 20 mM DTT, 150 μl mQ H_2_O) and heated for 8 min at 95° C. Cell debris were spun down (15.000 g, 5 min) and 14 µl of each sample were loaded on a SDS-PAGE gel (BioRad Protean TGX, 4–20%, 16 min, 300 V). A fast blotting procedure was performed (BioRad Trans-Blot Turbo, TGX Turbo protocol) to a PVDF membrane (Amersham Hybond PVDF). Three different antibodies were used in separate immunoblots directed towards the YFP split YFP variants (anti YFP_C_: Roche Diagnostics #11814460001; anti YFP_N_: BioLegend #902601), respectively, using a Pierce Fast Western Blot kit (ECL substrate) from Thermo Scientific. The chemiluminescence signal was detected via luminol enhancement from the kit and detection was manually performed and captured using a ChemiDoc MP imager from BioRad and Image Lab software.

### Fluorescence microscopy and image analysis

To confirm intracellular production of the BiFC complexes, cells of an overnight cultures of the different constructs were diluted to OD_600_ = 0.2 into 50 ml Falcon tubes and incubated at 30° C to reach an OD_600_ of 0.5. Cells were spun down at 3000 g for 5 min and resuspended in 500 μl H_2_0. The cell suspension was used to acquire the fluorescence images.

Images of the transformants were generated with an exposure time of 200 ms at an inverted Zeiss Axio Observer Z1 fluorescence microscope with an Axiocam 506 camera. A Plan-Apochromat 100x/1.40 oil DIC M27 objective was fitted to the microscope. YFP excitation was performed at 508 nm, using a 450–490 nm filter with a HXP mercury short-arc lamp. The emitted fluorescence light at 524 nm was analysed after passing a 500–550 nm filter. Zeiss Zen blue software was used to capture and process the data. For each construct, at least five different field of views (FOVs) were evaluated by moving the stage to obtain non-overlapping images, and the FOVs were randomly selected to avoid bias. A comparison with the single plasmid control YFP_C_-CaM was performed to distinguish signals from the background signal. Broken cells were not included in the data analysis. The imaging was repeated at least twice.

### Statistical analysis

Statistic details for the experiments performed can be found in the legend of Fig. 5. An unpaired two component t-test with Welch’s correction was used to determine statistical difference between the control groups (*p* < 0.0001).

## Supplementary Information

Supplementary Figure 1.
