# Supplementary figures and images for: A bimolecular fluorescence complementation flow cytometry screen for membrane protein interactions
Source: Sci Rep. 2021 Sep 28;11:19232. doi: 10.1038/s41598-021-98810-2 (PMC8478939; doi:10.1038/s41598-021-98810-2)

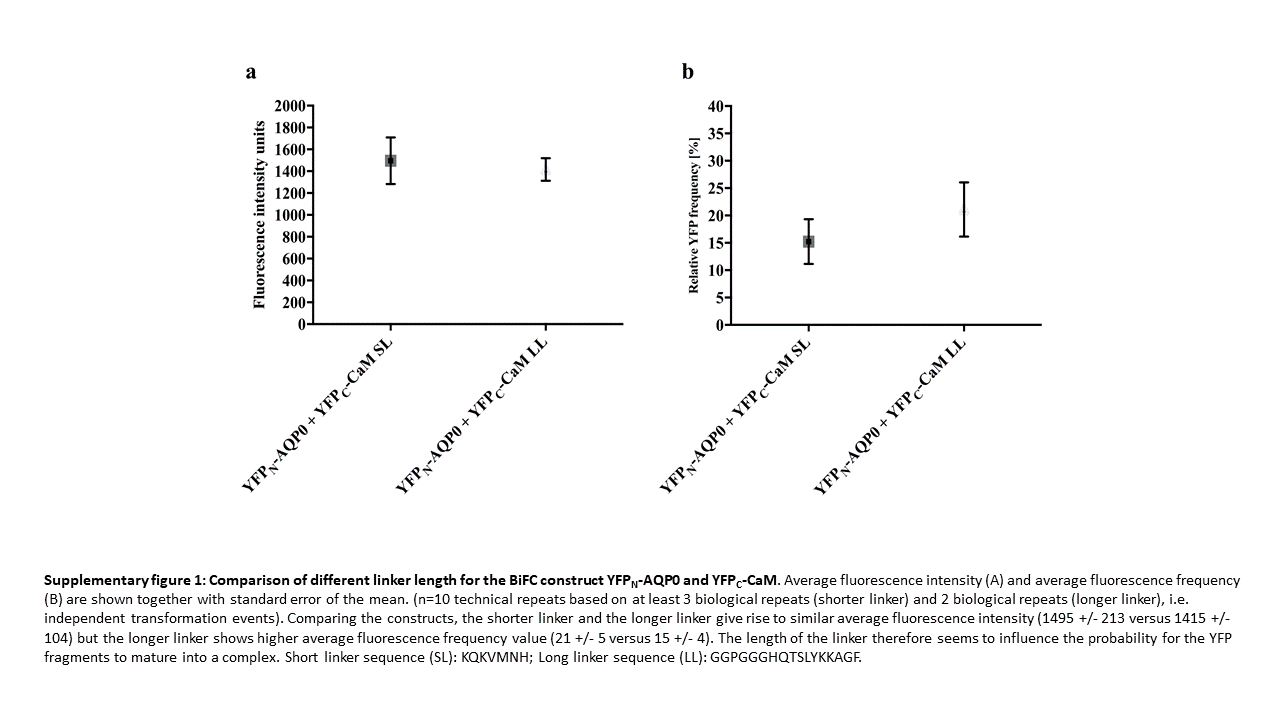

Supplement: Supplementary file 1 — Supplementary Figure 1. [file 41598_2021_98810_MOESM1_ESM.png]
